# Intrinsic differences in the mechanisms of Tie2 binding to angiopoietins exploited by directed evolution to create an Ang2-selective ligand trap

**DOI:** 10.1016/j.jbc.2021.100888

**Published:** 2021-06-18

**Authors:** Neil Bate, James Lodge, Nicholas P.J. Brindle

**Affiliations:** Department of Molecular & Cell Biology, and Department of Cardiovascular Sciences, University of Leicester, Leicester, United Kingdom

**Keywords:** directed evolution, receptor, angiopoietin, Tie2, cardiovascular, endothelial cell, angiogenesis, Ang2, angiopoietin-2, FACS, fluorescence-activated cell sorting, FBS, fetal bovine serum, Ig, immunoglobulin, Wt, wild-type

## Abstract

Angiopoietins Ang1 and Ang2 are secreted ligands for the endothelial receptor tyrosine kinase Tie2 essential for vascular development and maintenance. Ang1 acts as an agonist to maintain normal vessel function, whereas Ang2 acts as a Tie2 antagonist. Ang2 is increased in macular edema, sepsis, and other conditions, in which it blocks Ang1-mediated signaling, causing vascular dysfunction and contributing to disease pathology. Therefore, Ang2 is an attractive therapeutic target. Previously, we reported a Tie2 ectodomain variant that selectively binds Ang2 and acts as soluble ligand trap to sequester Ang2; however, the mechanism of Ang2-binding selectivity is unknown. In the present study, we used directed protein evolution to enhance Ang2-binding affinity of this Tie2 ectodomain trap. We examined contributions of individual residues in the ligand-binding interface of Tie2 to Ang1 and Ang2 binding. Surprisingly, different combinations of Tie2 residues were found to bind each ligand, with hydrophobic residues binding both ligands and polar residues contributing selectively to either Ang1 or Ang2 binding. Our analysis also identified a single Tie2 residue, His168, with a pivotal role in both Ang1 and Ang2 binding, enabling competition between binding ligands. In summary, this study reports an enhanced-affinity Ang2-selective ligand trap with potential for therapeutic development and reveals the mechanism behind its selectivity. It also provides the first analysis of contributions of individual Tie2 residues to Ang1 and Ang2 binding and identifies selectivity-determining residues that could be targeted in the future design of small molecule and other inhibitors of Ang2 for the treatment of vascular dysfunction.

Angiopoietin-2 (Ang2) is a secreted ligand whose expression is increased markedly in several pathologies, including sepsis, age-related macular degeneration, and cancer, where it contributes to disease progression (1, 2). The ligand binds the endothelial receptor tyrosine kinase Tie2 (3). This receptor also binds Ang1, a related ligand that is constitutively expressed by perivascular cells (4). Ang1 acts through Tie2 to protect the vasculature by promoting microvessel survival, inhibiting vascular inflammation and remodeling, and preventing leakage (1, 5). In inflammatory, and other conditions, the elevated concentration of Ang2 competes with Ang1 and acts to antagonize its protective effects, promoting endothelial activation, remodeling, and contributing to disease pathology (1, 6). This competition between Ang1 and Ang2 for Tie2 binding is a crucial regulatory mechanism controlling blood vessel quiescence and response to inflammation (1, 2).

There is considerable interest in developing inhibitors of Ang2 for therapeutic use. A number of monoclonal antibodies selectively binding Ang2 are in clinical trials for diseases ranging from COVID-19-induced acute respiratory distress to diabetic eye disease (7, 8). An alternative and complementary approach to antibodies for blocking pathological effects of ligands is the ligand trap (9). These traps comprise of a soluble receptor ectodomain fragment that binds the target ligand preventing it from eliciting its effects. Examples of ligand traps in clinical use include Etanercept, a soluble version of tumour necrosis factor-α receptor for treatment of rheumatoid arthritis, and Aflibercept, a vascular endothelial growth factor receptor fusion protein used to treat macular degeneration and cancer (10). A soluble form of the Tie2 ectodomain could act as a ligand trap. However, a major limitation of using the wild-type receptor is that it binds Ang1 with a similar or higher affinity as Ang2 (3, 4). Blocking Ang1 is highly undesirable as it is a protective ligand important in maintaining a functional vasculature (1). Therefore, creating an Ang2-selective ligand trap would require modifying the Tie2 ectodomain to prevent it binding and blocking the protective ligand Ang1, while maintaining its ability to bind Ang2.

The extracellular domain of Tie2 is comprised of two immunoglobulin-like (Ig) domains at the amino terminus, followed by three epidermal growth factor domains, a third Ig domain, and three fibronectin III repeats (11, 12, 13, 14). Crystal structures of Tie2 complexed with either Ang1 or Ang2 have been solved and show that both angiopoietins bind the second Ig domain of the receptor (15, 16). Comparison of the structures of Ang1:Tie2 and Ang2:Tie2 reveals that the two angiopoietins occupy the same position on the Tie2 Ig2 domain (15, 16). In the angiopoietins the receptor-binding site is located at the carboxy-termini of the ligands, within a fibrinogen-related domain (15, 16, 17). There are 13 amino acid residues in each of the angiopoietins located at the interface with Tie2 (15, 16). Of these, six are identical between Ang1 and Ang2, two residues are similar, and the remaining five residues differ.

Modifying Tie2 ectodomain for selective Ang2 binding is challenging as Tie2 is thought to bind Ang1 and Ang2 by a similar mechanism (15, 16). Furthermore, the contributions of individual amino acid residues at the ligand-binding site of Tie2 to Ang1 *versus* Ang2 binding are not known, making rational modification of this site extremely difficult. In a recent study, therefore, we used directed protein evolution to create a Tie2 ectodomain variant that binds Ang2 without binding Ang1 (18). In the present work we evolve this variant further to improve selective Ang2-binding affinity. To gain insight into the mechanism for selective binding of the variant, we determine the contribution of residues in the ligand-binding site of Tie2 to Ang1 or Ang2 binding. Surprisingly, this reveals significant differences in the molecular interactions used by Tie2 to bind each ligand. These differences were exploited in the evolution, resulting in the Ang2-selective variants. Moreover, our data identify His168 in Tie2 as having a pivotal role in enabling competition between Ang1 and Ang2 for Tie2 binding, highlighting this particular residue as crucial for the natural agonist:antagonist regulatory mechanism by which Tie2 controls vascular quiescence and inflammation.

## Results

### Improving affinity of an Ang2-selective Tie2 ligand trap

In previous work we used directed protein evolution to derive a Tie2 ectodomain variant with selective binding for Ang2 (18). In the present study we sought to increase affinity of this variant for Ang2 by iterative rounds of mutation and then selection with decreasing concentrations of Ang2. This was done using the method we described previously that combines cell surface display with exogenous gene diversification by somatic hypermutation in DT40 cells (18). In this approach Tie2 ectodomain incorporated into the rearranged immunoglobulin locus of DT40 cells undergoes mutagenesis by somatic hypermutation. The resulting ectodomain mutants are displayed on the cell surface, with each cell expressing multiple copies of a single ectodomain variant. Cells expressing variants that show increased Ang2 binding can then selected.

**Figure 1:**
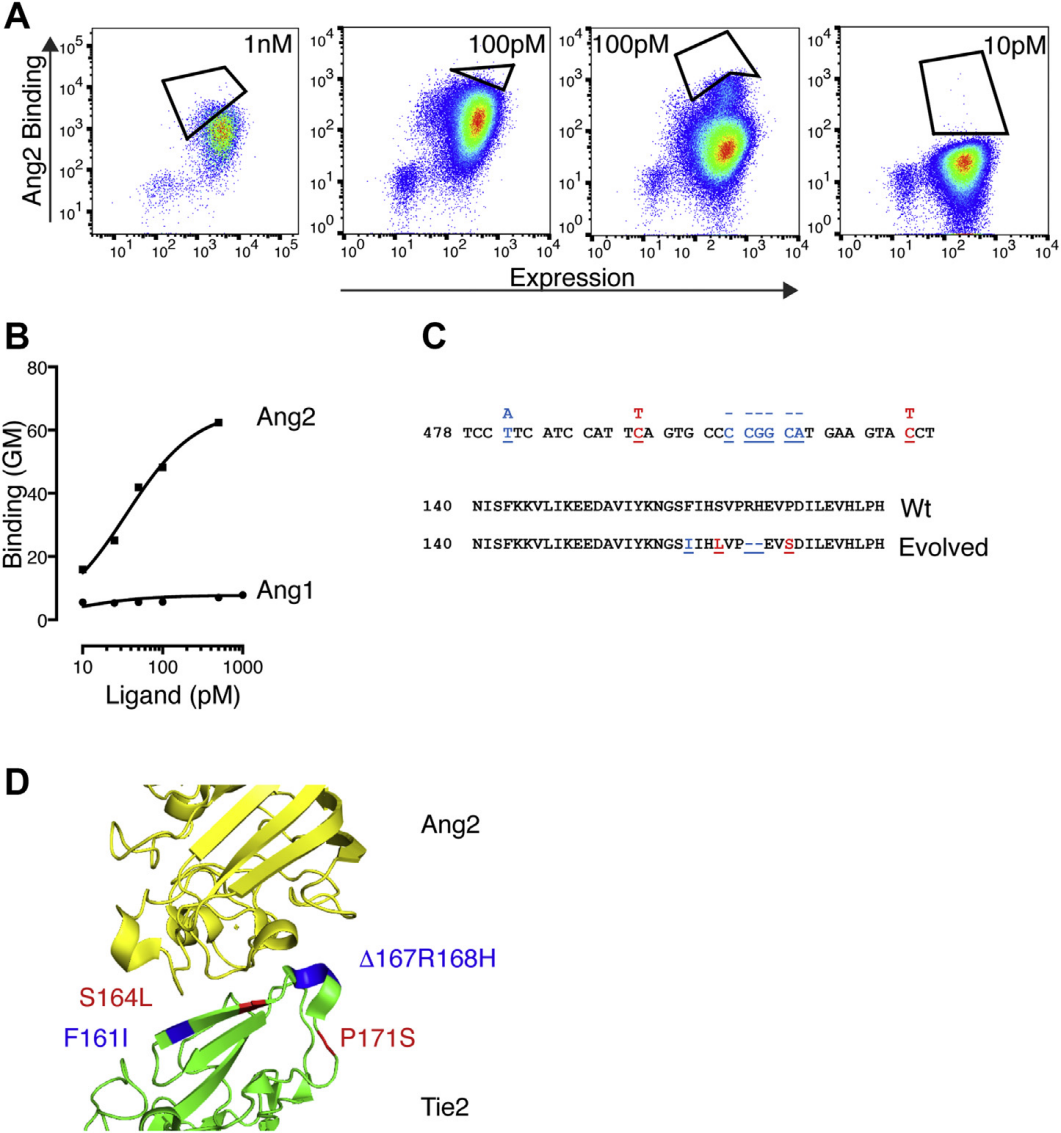
**Evolution of increased Ang2 affinity of ligand-selective Tie2 ectodomain.***A*, an Ang2-selective Tie2 variant (18) was evolved for enhanced Ang2 binding by somatic hypermutation and cell surface display in DT40 cells. FACS plots are shown of DT40 cells following binding of biotinylated-Ang2 and staining with streptavidin and anti-FLAG (detecting the epitope tag on Tie2 ectodomain to control expression level) followed by fluorescent secondary antibody. Sort windows are indicated for the four rounds of selection and diversification, selecting at each round for highest Ang2 binding. Selections were performed at progressively decreasing concentrations of Ang2, indicated for each sort. *B*, evolved Tie2 retains selectivity for Ang2. DT40 cells from the final selection were incubated with increasing concentrations of Ang1 and Ang2, as indicated, and bound ligand detected by immunostaining. Expression level of Tie2 was determined by costaining with anti-FLAG. Geometric mean fluorescence of bound ligand normalized to Tie2 expression was plotted against concentration of ligand. Concentration-dependent binding of Ang2, but not Ang1, was observed. *C*, mutations in evolved Tie2 ectodomain. Genomic DNA was prepared from the DT40 cells selected at 10 pM and cDNA encoding the Tie2 ectodomain amplified by PCR. Full-length sequencing revealed two nucleotide changes compared with starting sequence. Position of nucleotide change is shown in red and the substituted nucleotide shown above (*upper sequence*). Amino acid sequence of the modified region of the evolved ectodomain compared with wild-type Tie2. Amino acid changes resulting from the mutations introduced in the current evolution are shown in *red*, blue residues are those that were already present in the Ang2-selective starting variant previously reported (*lower sequences*). *D*, amino acid changes in the evolved ectodomain are located at the binding interface. The positions of the amino acid changes are shown on the structure of Tie2 ectodomain in complex with Ang2 (PDB accession number 2GY7 (15)). Amino acid changes introduced in the current evolution are shown in *red*, blue residues are those already present in the Ang2-selective starting variant.

In order to increase the affinity of the Ang2-selective Tie2 ectodomain for its ligand, previously evolved DT40 cells expressing Ang2-selective Tie2 ectodomain (18) were incubated with 1 nM Ang2 and cells with highest binding selected by fluorescence-activated cell sorting (FACS). Tie2 ectodomain sequences were then diversified further by allowing selected cells to grow (approximately 7 days) and the expanded population subjected to another round of selection, at a lower Ang2 concentration, followed by expansion of the selected population to diversify the Tie2 ectodomain sequence further. A total of four rounds of selection and diversification were performed at progressively decreasing Ang2 concentrations, from 1 nM to 10 pM (Fig. 1*A*). The cells selected at 10 pM were then tested for Ang2 and Ang1 binding by incubation of parallel populations of the selected cells with Ang1 or Ang2, staining for bound ligand, and measurement of mean fluorescence by flow cytometry (Fig. 1*B*). This confirmed the further evolved selected cells retained selective binding to Ang2.

The region encoding Tie2 ectodomain was recovered, by PCR, from genomic DNA extracted from the population of cells selected at 10 pM. This PCR product was sequenced and revealed a common set of mutations that result in substitution of Phe161 with Ile, deletion of Arg167 and His168 (these mutations were present in the starting population and give rise to Ang2 selectivity), as well as the additional substitutions of Ser164 with Leu, and Pro171 with Ser (Fig. 1*C*). Examination of the crystal structure of Tie2 complexed with Ang2 revealed all mutations map to the Tie2:Ang2-binding interface, with the exception of Pro171Ser (Fig. 1*D*).

**Figure 2:**
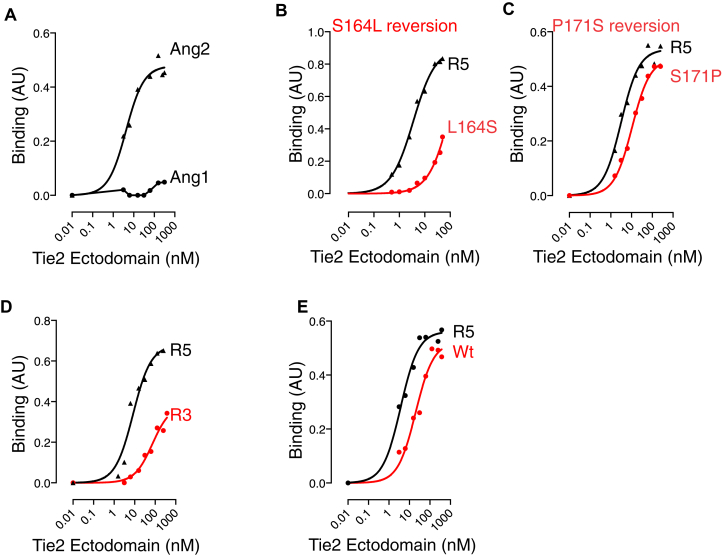
**Enhanced affinityAng2-selective Tie2 ectodomain**. *A*, binding of soluble evolved ectodomain to Ang1 and Ang2 measured by ELISA. Purified soluble Tie2 ectodomain fused with human Ig domain was analyzed for binding to immobilized Ang1 or Ang2 as indicated. Data are shown for a representative ELISA. *B*, Ser164Leu substitution contributes to Ang2 binding. Evolved ectodomain in which Leu164 was reverted back to Ser was tested for binding to Ang2. Reversion to wild-type Ser decreased affinity of binding. *C*, Pro171Ser substitution makes a minor contribution to Ang2 binding. Evolved ectodomain in which Ser171 was reverted back to Pro was tested for binding to Ang2. *D*, evolved ectodomain binds Ang2 with higher affinity than R3. Evolved ectodomain and starting Ang2-selective ectodomain (R3) were analyzed for binding to Ang2. All data shown are individual binding curves representative of at least two independent experiments. *E*, comparison of binding of Wt-Tie2 ectodomain (monomeric) with evolved ectodomain-Fc (R5).

The evolved Tie2 ectodomain, with the mutations described above, was cloned into an expression vector as a fusion construct with human Ig Fc domain. This Tie2 ectodomain-Fc with Phe161Ile, deletion of Arg167 and His168, Ser164Leu and Pro171Ser was designated R5. Following transfection into Hek293 cells, the expressed soluble R5 protein was purified and used in binding assays. Wild-type and R5 proteins expressed to similar levels. Binding to Ang1 and Ang2 was assessed by enzyme-linked immunosorbent assay (ELISA). Consistent with findings on the DT40 cells, the newly derived Tie2 ectodomain variant is selective for Ang2 binding (Fig. 2*A*). In order to test whether the two new substitutions, Ser164Leu and Pro171Ser, contribute to binding of the ectodomain variant, we examined the effects of reverting each position back to the original residue. Reversion of Leu164 back to Ser resulted in a marked decrease in binding to Ang2 (Fig. 2*B*). These data confirm Ser164Leu as a key contributor to binding. In contrast, changing Ser171 back to Pro decreased Ang2 binding, but to a much lesser extent than the Ser164 reversion (Fig. 2*C*). Pro171 is not located in the Ang-binding interface of Tie2, though is close to it (Fig. 1*C*), and it is possible the subtle effect of the Pro171Ser substitution reflects an indirect effect on binding, such as increased local stability. The R5 Tie2 ectodomain variant was compared with the parent Ang2-selective Tie2 ectodomain (R3) described previously (18). Consistent with the selection strategy, R5 was found to have substantially increased binding to Ang2, compared with parent ectodomain (Fig. 2*D*). R5 therefore offers a significant improvement over the original Ang2-selective Tie2 ectodomain for potential development as a ligand trap. Commonly, ligand traps are used as dimeric fusion proteins (9). We therefore compared the evolved R5 ectodomain as a dimeric Fc fusion protein with the monomeric wild-type (Wt) form of Tie2 that the ligand trap would normally compete with for Ang2 binding (Fig. 2*E*). R5-Fc showed more than 6-fold higher relative affinity than monomeric Tie2 for Ang2 binding (EC50 of 3.40 ± 0.41 nM for R5-Fc *versus* 22.72 ± 4.25 nM for Wt monomer, mean, and SEM for three experiments).

### Roles of residues in the Tie2 Phe161-Glu169 motif in binding Ang1 and Ang2

**Figure 3:**
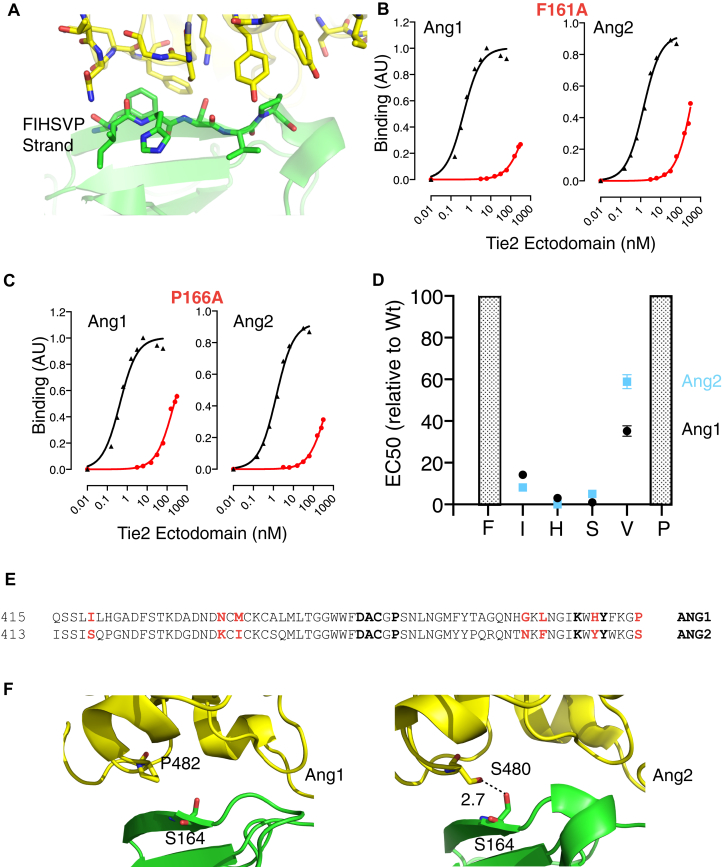
**Phe161 and Pro166 in Tie2 are dominant contributors to both Ang1 and Ang2 binding**. *A*, the position of the Phe161-Pro166 strand in Tie2 is highlighted in the structure of Tie2 (*green*) in complex with Ang2 (*yellow*). PDB accession number 2GY7 (15). *B*, representative binding plots are shown for Phe161A and (*C*) Pro166A Tie2 ectodomains to Ang1 and Ang2. Wt binding curves are depicted in *black* and mutant binding curves in *red*. *D*, the change in EC50 for binding to Ang1 and Ang2 of Tie2 with substitution of Ala for each position in the Phe161-Pro166 strand is shown relative to Wt-Tie2. Relative changes were calculated as mutant EC50/Wt EC50 determined within the same experiment and are shown as means and SEM for at least three independent experiments. Shaded columns for Phe161 and Pro166 indicate increase in EC50 greater than measurable in these assays. *E*, alignment of part of the Ang1 and Ang2 sequences that encompass the residues located at the Tie2 interface. Residues at the interface are shown in *bold*, those interface residues that differ between Ang1 and Ang2 are in *red*. *F*, the structure of Tie2 ectodomain in complex with Ang1 (PDB accession number 4K0V (16)) and Ang2 (PDB accession number 2GY7 (15)) is shown highlighting the residues interacting with Ser164. Potential interactions are indicated by *dashed lines* and estimated distances are indicated in *Angstroms*. Tie2 is in green and angiopoietins in *yellow*.

In order to gain insight into the mechanisms for selective Ang2 binding of R5 (and its parent, R3), we assessed the contributions of each amino acid in the 161Phe-169Glu motif of wild-type Tie2 (Fig. 3*A*). This is the motif in which the mutations selected by directed evolution were focussed. To do this wild-type-Tie2 ectodomain-Fc and a series of mutant Tie2-ectodomain-Fc fusion proteins were constructed with Ala substitution at each of the positions from 161Phe to 169Glu. Fusion proteins were expressed, purified, and mutants and wild-type were compared in assays of binding to Ang1 and Ang2. As shown in Figure 3, *B* and *C*, Phe161 and Pro166 have major roles in both Ang1 and Ang2 binding. Substituting Ala for either Phe161 or Pro166 suppressed Ang1 and Ang2 binding dramatically. Indeed, concentrations of protein required for maximal binding were beyond those that could be incorporated in the binding assay, preventing calculation of the half-maximal binding concentrations for Ang1 and Ang2 binding to these two mutants. These data indicate that Phe161 and Pro166 make major contributions to binding Ang1 and Ang2.

Binding analysis of the other mutant ectodomain fusion proteins reveals that Ile162, His163, and Val165 all contribute to Ang binding (Fig. 3*D*), with Ala substitution causing a decrease in Ang1 and Ang2 binding to a similar degree (Fig. 3*D*). Of these residues, substitution of Val165 caused the biggest decrease in Ang binding, with around a 30-fold decrease in relative affinity compared with Wt-Tie2 for Ang1 and Ang2 binding. Substitution of Ile162 caused an approximate 10-fold decrease in Ang1 and Ang2 binding, while His163 substitution decreased binding by 3-fold (Fig. 3*D*). Interestingly, Ser164 makes little contribution to Ang1 binding, as substitution with Ala did not affect relative binding affinity for Ang1 compared with wild-type Tie2. However, Ser164 certainly contributes to Ang2 binding, with an S164A substitution causing an approximate 7-fold decrease in relative binding affinity for Ang2 compared with wild-type Tie2 (Fig. 3*D*). To gain insight into how Ser164 could contribute to Ang2 but not Ang1 binding, differences between Ang1 and Ang2 structures were examined. Barton *et al.* (15, 16) report there are 13 residues in Ang1 and Ang2 that are located at the interface with Tie2, and seven of these differ between the two ligands (Fig. 3*E*). The structure of Ang2 in complex with Tie2 reveals Ser164 is located close enough to Ser480 in Ang2 to form a hydrogen bond (Fig. 3*F*). However, in Ang1 the position corresponding to Ang2 Ser480 is occupied with Pro and therefore unable to hydrogen bond with Ser164 (Fig. 3*F*). This difference between Ang1 and Ang2 in ability to hydrogen bind with Ser164 likely underlies the differential contribution of Ser164 to Ang2 *versus* Ang1 binding.

**Figure 4:**
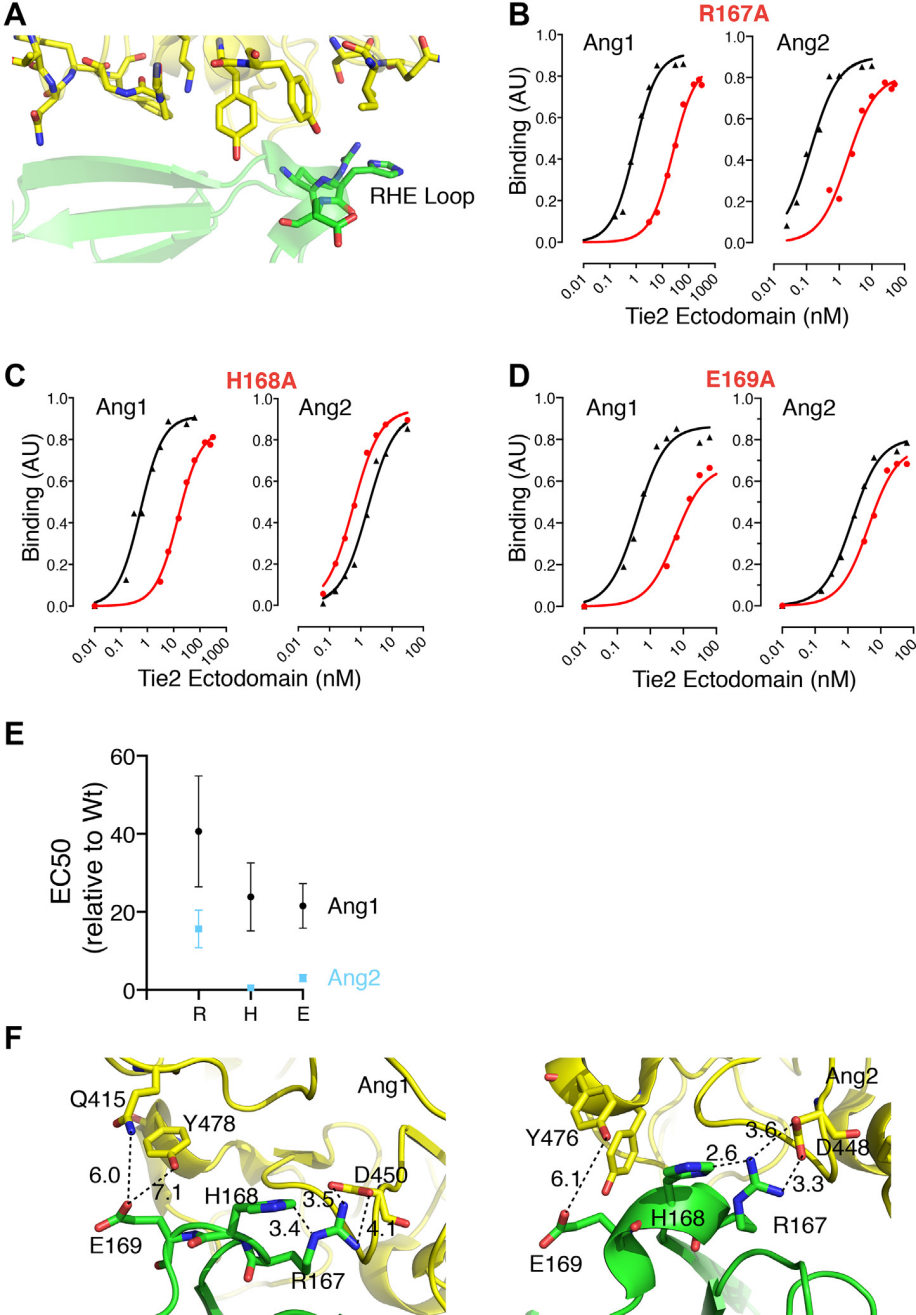
**The Arg167-Glu169 loop in Tie2 preferentially contributes to Ang1 binding**. *A*, the position of the Arg167-Glu169RHE loop in Tie2 is highlighted in the structure of Tie2 (*light green*) in complex with Ang2 (*yellow*). PDB accession number 2GY7 (15). Wt and Ala mutants of Tie2 ectodomains were analyzed for binding to immobilized Ang1 and Ang2 by ELISA. Representative ELISA plots are shown for binding of (*B*) Arg167Ala, (*C*) His168Ala, and (*D*) Glu169Ala Tie2 ectodomains to Ang1 and Ang2. Wt binding curves are depicted in *black* and mutant binding curves in *red*. *E*, the change in EC50 for binding to Ang1 and Ang2 of Tie2 with substitution of Ala for Arg167, His168, and Glu169 is shown relative to Wt-Tie2. Relative changes were calculated as mutant EC50/Wt EC50 determined within the same experiment and are shown as means and SEM for at least three independent experiments. *F*, the structure of Tie2 ectodomain in complex with Ang1 (PDB accession number 4K0V (16)) and Ang2 (PDB accession number 2GY7 (15)) is shown highlighting the residues interacting with the Arg167-Glu169 loop. Potential interactions are indicated by *dashed lines* and estimated distances are indicated in *Angstroms*. Tie2 is in *green* and angiopoietins in *yellow*.

Residues Arg167, His168, and Glu169 form a loop at the end of the 161Phe-166Pro strand (Fig. 4*A*), and the contribution of this loop to Ang binding was analyzed. As shown in Figure 4, *B* and *E*, Tie2(H168A) displayed an approximate 30-fold decrease in Ang1 binding and, surprisingly, a 2-fold increase in Ang2 binding relative to Wt-Tie2. Substitution of Arg167 or Glu169 with Ala decreased binding to both Ang1 and Ang2. Specifically, Arg167Ala caused a 35-fold decrease in Ang1 binding compared with a 9-fold decrease in Ang2 binding (Fig. 4, *C* and *E*), and Glu169Ala caused a 12-fold decrease in Ang1 compared with a 3.7-fold decrease in Ang2 binding (Fig. 4, *D* and *E*), relative to Wt-Tie2. Substitutions in the Arg167-Glu169 loop, therefore, decreased the ability of Tie2 to bind Ang1 to a far greater extent than they affected Ang2 binding (Fig. 4*E*). The structures of Tie2 in complex with Ang1 or Ang2 were examined to gain insight into the preferential binding of Ang1 to the Arg167-Glu169 loop (Fig. 4*F*). This reveals that Arg167 in Tie2 is able to interact with Asp448 in Ang2 and the corresponding Asp450 in Ang1 (Figs. 3*E* and 4*F*). However, there are differences in the position of Asp448 in the Ang2 structure compared with Asp450 in Ang1 structure, and interaction with Asp448 in Ang2 brings the position of Arg167 into conflict with His168 in Tie2 (Fig. 4*F*). This clash may compromise the contribution of Arg167 to Asp448 binding in Ang2, leading to the lesser contribution of Arg167 to Ang2 binding than to Ang1 binding. The clash between Arg167 and His168 in Tie2 complexed with Ang2 may also contribute to the inhibitory effect of His168 on Ang2 binding, with loss of the His side chain relieving the clash and increasing binding to Ang2 by allowing better access of Arg167 to Ang2 Asp448. Our data clearly show that Glu169 contributes to both Ang1 and Ang2 binding, with a greater contribution to Ang1 binding. The structure of Tie2 complexed with Ang1 shows that the closest residues to Glu169 are Gln415 and Tyr478 (Fig. 4*F*). Gln415 was not identified originally as an interfacial residue (15, 16). However, the resolution of the structure makes ascribing a precise position to the Glu169 side chain difficult, and it is conceivable that both Gln415 and Tyr478 in Ang1 could be within hydrogen bonding distance of Glu169. In contrast, the Gln415 position is occupied by Ile in Ang2 and would be unable to contribute to hydrogen bonding with Glu169, and this may explain the lesser contribution of Glu169 to Ang2 binding that we observe.

Together, these data demonstrate that hydrophobic residues in the Tie2-binding interface, specifically Phe161, Ile162, Val165, and Pro166, make substantial contributions to Ang1 and Ang2 binding. Where measurable, these residues appear to contribute to a similar extent to both Ang1 and Ang2 binding. In contrast, the Arg167-His168-Glu169-loop is much more selective for Ang1 than Ang2 binding. Furthermore, residue His168 makes a substantial contribution to Ang1 binding but is inhibitory for Ang2 binding. Overall Ang1 and Ang2 binding is similarly dependent on the hydrophobic residues at the Tie2-binding interface, whereas the polar residues contribute selectively to Ang1 and Ang2 binding.

### Mechanism for Ang2 selectivity

**Figure 5:**
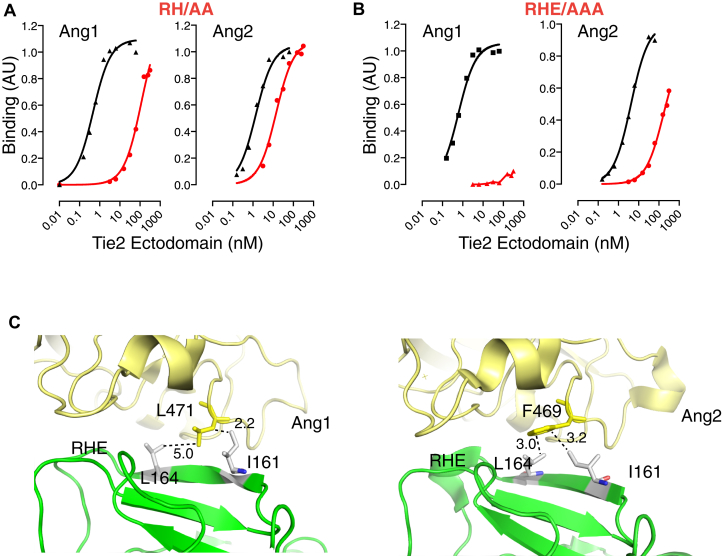
**Mechanism for Ang2-selective binding.***A*, effects of substituting Arg167 and His168 with Ala on binding to Ang1 and Ang2. *B*, effects of substituting Arg167, His168, and Glu169 with Ala on binding to Ang1 and Ang2. *C*, the structure of Tie2 ectodomain in complex with Ang1 (PDB accession number 4K0V (16)) and Ang2 (PDB accession number 2GY7 (15)) is shown in which Phe161 is substituted by Ile and Ser164 substituted by Leu. Potential hydrophobic interactions of Ile161 and Leu164 are shown by *dashed lines*. Estimated distances are indicated in *Angstroms*. Side chains of substituted Tie2 residues are shown in *gray* and of angiopoietins in *yellow*. The position of the RHE-loop is also indicated.

The contributions of Tie2 residues Phe161-Glu169 to binding Ang1 and Ang2 provide insights into the molecular basis for the selectivity of the ectodomain variants derived by directed evolution. Notably, we find that Arg167 and His168 make a substantial contribution to Ang1 binding and much lower contributions to Ang2 binding, with His168 actually inhibiting Ang2 binding (Fig. 4). Directed evolution for Ang2 selectivity resulted in deletion of both Arg167 and His168 (18). Based on the data in Figure 4, loss of His168 would be expected to decrease Ang1 binding and increase Ang2 binding, whereas deletion of Arg167 would decrease the relative affinity of Tie2 35-fold for Ang1 but only 9-fold for Ang2. Overall, loss of Arg167 and His168 side chains therefore would be expected to cause a much greater decrease in affinity of Tie2 for Ang1 than for Ang2, favoring Ang2 selectivity. To test this, we created a double substitution in Tie2 ectodomain of Arg167Ala and His168Ala and tested binding to Ang1 and Ang2. As predicted, the double substitution decreased binding to Ang1 to a much greater extent than binding to Ang2 (Fig. 5*A*). However, there is still clear binding to Ang1, in contrast to what is observed for R5. In this context, it should be noted that whereas the Ala substitutions indicate effects of loss of the Arg167 and His168 side chain contributions to binding, in the evolved variant Arg167 and His168 are actually deleted. This deletion would be expected to both remove these side chain contributions to binding and reconfigure the structure in the RHE loop due to loss of the Arg and His backbones. One consequence of this is likely to be repositioning of Glu169, preventing it from participating in its normal interaction with ligand. As indicated in Figure 4, *D* and *E*, loss of the Glu169 side chain decreases Tie2 affinity approximately 3-fold more for Ang1 relative to Ang2, which would be expected to further decrease Ang1 binding to a greater extent than Ang2 binding. We therefore tested the effect of the additional removal of the Glu169 side chain by alanine substitution in the Arg167Ala/His168Ala Tie2 ectodomain mutant. Indeed, loss of Arg167/His168 and Glu169 side chains resulted in negligible Ang1 binding while retaining clear, albeit reduced, binding to Ang2 (Fig. 5*B*). These findings support the notion that deletion of Arg167 and His168 in the evolved Tie2 ectodomain causes the preferential loss of Ang1 binding. This involves the direct effects of loss of Arg167/His168 side chains and likely secondary effects of preventing Glu169 from contributing to ligand binding.

While the changes in the RHE loop in the evolved Ang2-selective Tie2 variant can explain loss of Ang1 binding, the Phe161Ile and Ser164Leu substitutions contribute to the Ang2 binding of the evolved ectodomain. Examination of the structure of wild-type Tie2 in complex with Ang2 reveals that both an Ile at position 161 and a Leu at position 164 in Tie2 could contribute to a hydrophobic interaction with Phe469 in Ang2 (Fig. 5*C*). In contrast, Ang1 has a Leu residue (Leu471) in the position corresponding to Ang2 Phe469. This Leu471 is poorly positioned to interact productively with Tie2 Ile161, where it may clash due to proximity. Furthermore, the distance between Leu471 in Ang1 and Tie2 Leu164 is around 5 Å, compared with 3 Å between Tie2 Leu164 and Ang2 Phe469 (Fig. 5*C*). Together, this would favor Ang2 binding over Ang1 binding.

It should be noted that the putative residue interactions between ligands and mutant Tie2 discussed above are based on the structures of Wt-Tie2 in complex with Ang1 or Ang2 (15, 16). A more accurate and definitive analysis of residue interactions between angiopoietins and ectodomain variants will require solving the structure of each ligand complexed with the Tie2 variants.

### His168 has a pivotal role in enabling competition between Ang1 and Ang2

**Figure 6:**
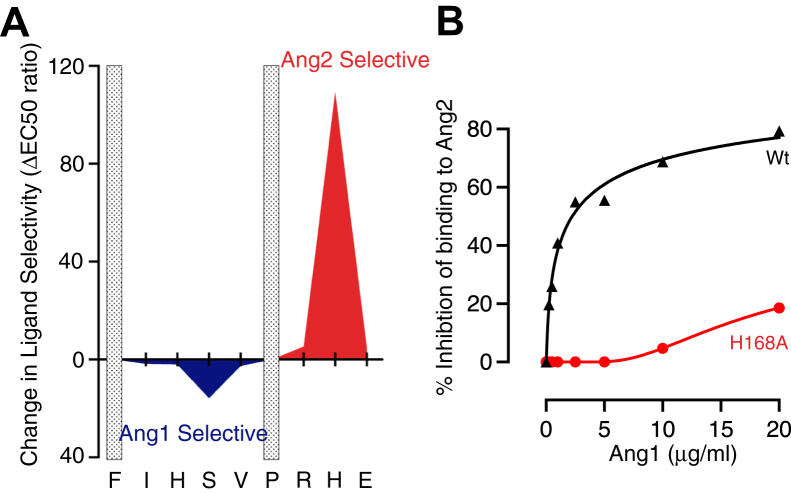
**Competitive binding of Ang1 and Ang2 to Tie2 is critically dependent on His168.***A*, effects of Ala substitution at each position in Tie2 Phe161-Glu169 motif on selectivity of ectodomain binding for Ang1 and Ang2. Selectivity is expressed as ratio of EC50 for each ligand, Ang2 selectivity shown as above *x*-axis and Ang1 selectivity below. *B*, binding of Tie2 His168Ala to Ang2 is resistant to Ang1 competition. Wt and His168Ala Tie2 ectodomains were bound to Ang2 immobilized on ELISA plates and the ability of increasing concentrations of Ang1 to displace Tie2 was determined. Data are plotted as percentage inhibition of Tie2 binding to Ang2. A representative concentration curve is shown.

The ability of Ang1 and Ang2 to compete for binding to Tie2 is essential for regulation of Tie2 signaling in normal physiological conditions and for controlling vascular responses during new blood vessel formation and in pathological situations such as inflammation. Effective competition between the ligands requires Ang1 and Ang2 to bind with similar affinities to their receptor and the ratio of Ang2:Ang1 affinities is close to 1 for Wt-Tie2 (3). Our data show that relative binding affinities of Tie2 for Ang1 and Ang2 are dramatically influenced by residue position His168, with loss of the His side chain causing more than a 100-fold shift in binding selectivity of Tie2 in favor of Ang2 (Table 1, Fig. 6*A*). This selectivity switch would be expected to suppress the ability of Ang1 to compete with Ang2 for binding on Tie2. To test this, Tie2(H168A) and wild-type Tie2 were tested for their ability to bind Ang2 in the presence of increasing concentrations of Ang1. As expected, Ang1 was able to compete effectively with Ang2 for binding to wild-type Tie2 (Fig. 6*B*). In contrast, the Tie2(H168A) mutant was found to be resistant to competition by Ang1, with 20 μg/ml Ang1 causing a 25.7 ± 3.9% inhibition (mean and SEM, n = 3) of binding to Ang2, in comparison to the 0.3 ± 0.1 μg/ml Ang1 (mean and SEM, n = 3) required to cause a similar degree of inhibition of Wt-Tie2 binding. These data demonstrate that His168 is a crucial residue enabling competition between Ang1 and Ang2.

**Table 1:** Summary of effects of Ala substitutions in the Tie2 Phe161-Glu169 motif on binding to Ang1 and Ang2

Tie2	EC50 (nM)	
	Ang1	Ang2
Wt	0.66 ± 0.08	1.37 ± 0.19
F161A	ND	ND
I162A	6.38 ± 0.38	23.06 ± 2.88
H163A	1.38 ± 0.19	5.88 ± 0.94
S164A	0.44 ± 0.06	14.38 ± 1.44
V165A	14.31 ± 0.13	77.69 ± 3.5
P166A	ND	ND
R167A	26.56 ± 1.75	10.31 ± 0.69
H168A	26.38 ± 8.44	0.50 ± 0.06
E169A	9.00 ± 1.94	3.94 ± 0.63

Wt and Ala mutants of Tie2 ectodomains were analyzed for binding to immobilized Ang1 and Ang2 by ELISA. Concentrations of Tie2 ectodomains required for 50% of maximal binding (EC50) to Ang1 and Ang2 were derived from all the ELISA binding curves and are presented as means and SEM for between three and 20 independent experiments. EC50 for Phe161Ala and Pro166Ala could not be determined (ND) as Tie2 concentrations could not be increased sufficiently to reach saturation binding for these two mutants.

## Discussion

In this study we have used directed protein evolution to improve binding affinity of an Ang2-selective Tie2 ectodomain variant. Insights into the mechanism for Ang2 selectivity of this variant were then sought by analysis of the contribution of residues in the wild-type Tie2 ligand-binding interface to binding of Ang1 and Ang2. Surprisingly, we found important differences in the way in which Tie2 interacts with each ligand. In particular we identified residues and residue groups that contribute preferentially to either Ang1 or Ang2 binding. Furthermore, we found that directed evolution had exploited these same residue positions to create the Ang2 selective binding variants.

Ang2 acts as a pathological ligand in a range of diseases including sepsis, cardiovascular disease, and acute respiratory distress syndrome (1, 6). In these conditions Ang2 levels are increased and the ligand acts as an antagonist to block binding of Ang1 to its receptor, Tie2. Under normal conditions Ang1 is constitutively expressed and acts through Tie2 to maintain endothelial quiescence and suppress vascular inflammation and remodeling. Antagonism of Ang1 by Ang2 prevents these protective effects, leading to vascular activation, remodeling, and inflammation that contribute to pathology and disease progression in these conditions (1, 6). Blocking Ang2 action with neutralizing antibodies has been shown to suppress Ang2-mediated vascular effects and improve outcomes in preclinical models of a range of diseases, including sepsis, acute and chronic myocardial damage following infarction and inflammatory lung disease (19, 20, 21). Consequently, several monoclonal antibodies that block Ang2 are currently in clinical trials for conditions from acute respiratory distress syndrome induced by COVID-19 to diabetic eye disease (7, 8). In addition to antibodies, ligand traps have been successful in blocking effects of ligands whose elevated expression contributes to disease (9). For example, Etanercept, a ligand trap that targets TNFα, is used successfully in rheumatoid arthritis patients (10). There are significant advantages of ligand traps for blocking pathological effects of ligands. Traps can be smaller in size and have better tissue penetration compared with antibodies, as well as fewer problems with immunogenicity.

A ligand trap for Ang2 would provide an attractive complementary approach to the use of antibodies. However, the ability of Tie2 ectodomain to bind the protective ligand Ang1, as well as Ang2, is a major limitation to its development as a ligand trap. Blocking Ang1 action would have serious deleterious effects on the vasculature. Ang1 and Ang2 both bind to the same site in Tie2, and they are thought to use similar mechanisms of binding (15, 16). This makes rational modification of the ligand binding site of Tie2 ectodomain to create an Ang2-selective trap very challenging, particularly as the contribution of individual residues to relative binding to Ang1 and Ang2 was previously unknown. Hence, in a previous study, we used directed protein evolution to create an Ang2-selective ligand trap (18). This bound to Ang2 but not Ang1 and was able to selectively block Ang2 effects on cells. However, although the trap was selective for Ang2, its affinity for the ligand was relatively low. In addition, the mechanism for Ang2 selectivity was unknown. In the present study we developed this Ang2-selective variant by further directed evolution to increase binding affinity for Ang2. This resulted in a Tie2 variant with two additional mutations that caused a marked increase in binding affinity for Ang2 compared with the original Ang2-selective trap. As an Fc-fusion protein, the improved Tie2 variant binds Ang2 with higher affinity than wild-type Tie2 but, unlike wild-type receptor, does not bind Ang1. This enhanced affinity Ang2-selective Tie2 ectodomain represents an attractive candidate for further development into a therapeutic inhibitor of Ang2.

In order to understand why the evolved ectodomain is Ang2-selective, we analyzed the contribution of residues in the region of Tie2 that contained the mutations selected by the directed evolution, namely the Phe161-Glu169 motif. A key finding was that Arg167 and His168 in Tie2 are major contributors to Ang1 binding. In contrast, Arg167 contributes much less to binding of Ang2, and His168 actually inhibits Ang2 binding. Both Arg167 and His168 were deleted in evolution of Ang2-selective Tie2. This loss of Arg167 and His168 clearly favors Ang2 selectivity by removing the inhibitory effect of His168 for Ang2 binding and by decreasing Ang1 affinity by loss of both Arg167 and His168. This was confirmed by the effects of Arg167Ala together with His168Ala substitutions on Ang1 and Ang2 binding shown in Figure 5. Furthermore, our data is consistent with the deletion of Arg167 and His168 in the evolved Tie2 variant also affecting the ability of Glu169 to contribute to binding. Loss of Arg167 would also decrease affinity for Ang2, though considerably less than the decrease for Ang1, similarly loss of Glu169 would decrease Ang1 binding more than Ang2 binding. Presumably, the sacrifice in Ang2 affinity caused by Arg167 loss and functional loss of Glu169 was offset by the larger decrease in Ang1 affinity in order to satisfy the selective pressure for preferential Ang2 binding exerted in the evolution. Loss of Ang1 binding is therefore explained by changes in the RHE loop in the evolved ectodomain. The additional changes of Phe161Ile and Ser164Leu in the evolved ectodomain act to enhance Ang2 binding in the context of the loss of Arg167 and His168. Pro171 is located at the end of the RHE loop and its substitution with Ser in the evolved mutant enhances Ang2 binding (Fig. 2*C*). This may reflect stabilization of the end of the FIHSVP strand following reconfiguration of the RHE loop due to loss of Arg167 and His168. Alternatively, or in addition, the repositioning of the end of this loop could provide new binding opportunities for the substituted Ser with Ang2.

Analysis of the roles of residues in the Phe161-Glu169 motif also revealed unexpected differences in the mechanisms by which Tie2 binds Ang1 and Ang2. It had been thought that the receptor binds both ligands by a similar mechanism (15, 16). However, we found that while Phe161, Ile162, His163, Val165, and Pro166 all contribute similarly to Ang1 and Ang2 binding, Ser164 contributes preferentially to Ang2 binding, His168 makes a major contribution to Ang1 binding but inhibits Ang2 binding, and the Arg167-His168-Glu169 loop makes a much greater contribution to Ang1 than Ang2 binding. Thus, while binding of both ligands is dominated by hydrophobic residues in the Phe161-Glu169 motif, the polar residues contribute selectively to Ang1 or Ang2 binding. Tie2 therefore utilizes different combinations of molecular interactions to bind Ang1 and Ang2. It is noteworthy that directed evolution for preferential Ang2 binding resulted in mutations of the three residues that contribute most selectively to angiopoietin binding (Ser164, Arg167, His168). This identification of selectivity-determining residues in the Phe161-Glu169 motif may be useful in future efforts aimed at rational design of Tie2 variants with selective angiopoietin-binding characteristics and in creation of small-molecule inhibitors of Ang2 binding.

In the present study, ELISA has been used to measure binding and assess the impact of mutations relative to Wt binding. Additional depth of insight would be provided by determination of the effects of the mutations on binding kinetics. However, Ang1 exists as a mix of different oligomeric forms, as does Ang2 (22), and this prevents determination of binding kinetics by approaches such as surface plasmon resonance.

Our finding that His168 inhibits Ang2 binding to Tie2 was most unexpected and has implications for a key regulatory mechanism that controls vascular growth and inflammation. The ability of Ang1 and Ang2 to compete for the same binding site on Tie2 determines the signaling outcome of the receptor, with Ang1 activating Tie2 and promoting vascular quiescence and Ang2 able to act as a competing antagonist to promote vessel inflammation (1, 3). This competition for binding to Tie2 is fundamentally important for enabling the complex agonist:antagonist regulatory system that controls vascular quiescence and response to inflammatory and angiogenic conditions. Ang1 and Ang2 bind with similar affinity to the ligand-binding site on the Ig2 domain of Tie2 (23). Our finding that His168 has a pivotal effect on binding selectivity of Tie2 for Ang1 and Ang2 allowed us to test how a divergence in affinities for the two ligands would affect competition between the Ang1 and Ang2 for binding Tie2. We found that loss of the His side chain at position 168 in Tie2 severely limits the ability of Ang1 to compete with Ang2 for binding to Tie2. This competition is essential for normal regulation of vascular function. Thus, a fundamental mechanism for the control of vascular growth and inflammation in humans is critically dependent on the identity of the amino acid residue at position 168 of the receptor tyrosine kinase Tie2.

In summary, this study describes the evolution of a Tie2 variant with increased selective binding for the pathogenic ligand Ang2 that may be useful for development as an Ang2 ligand trap. Tie2 residues in the vicinity of those targeted by the evolution were examined for their contribution to Ang1 and Ang2 binding. This revealed that both angiopoietins utilize interaction with a common set of hydrophobic residues at the Tie2 ligand-binding interface; however, polar residues in this region contribute preferentially to binding either Ang1 or Ang2. Furthermore, directed evolution was found to have exploited the differential contributions of these polar residues to generate an Ang2-selective binder, notably, deleting the key Ang1-binding residues in the Arg167-His168-Glu169 loop. Finally, our data highlights the importance of His168 in allowing Ang1 and Ang2 to compete for Tie2 binding, thereby enabling a key regulatory mechanism controlling vascular function.

## Experimental Procedures

### Cell surface display and somatic hypermutation

Tie2 ectodomain was diversified and selected using the DT40 system that we have described previously (18). This method combines in-cell mutagenesis of exogenous genes, *via* somatic hypermutation, with display on the cell surface to allow selection of desirable variants. Briefly, cDNA encoding Tie2 ectodomain (residues 1–442) followed by a FLAG-tag and residues 514–562 of platelet-derived growth factor receptor β, which incorporates the transmembrane domain, was inserted into the pHypermut2 vector (24). This construct has been described in detail previously (18). DT40 cells were transfected by electroporation and stable transfectants established by growth in puromycin. DT40 clones in which the Tie2 construct was integrated into the rearranged Ig locus were identified by PCR and Tie2 ectodomain expression, surface expression, and angiopoietin binding confirmed by immunoblotting and immunostaining as described (18). Cells were cultured in RPMI containing 7% (v/v) fetal bovine serum (FBS) and 3% (v/v) chicken serum at 37 °C and 5% CO_2_.

Tie2 ectodomain variants with desired binding phenotypes were selected by FACS following ligand binding. Briefly, approximately 40 million DT40 cells were washed and incubated with ligand (as indicated in Results) in PBS with 10% (v/v) FBS for 30 min at room temperature. After washing bound angiopoietin was detected by immunostaining and fluorescent secondary antibodies. Cells were simultaneously stained with anti-FLAG to detect expression level of Tie2 ectodomain on individual cells. Stained cells were selected by FACS using the sort windows indicated in Results. Selected cells were resuspended in DT40 culture medium and expanded for subsequent selections.

### Sequencing

Initial sequencing was performed directly on PCR products from the selected cell populations. This involved isolation of genomic DNA from an aliquot of the selected population of cells using PureGene DNA isolation kit (Qiagen). Tie2 ectodomain was amplified by PCR, purified PCR products were then sequenced, and chromatograms examined directly. Individual Tie2 ectodomain variants were sequenced from final homogeneous cell populations by amplification of Tie2 ectodomain from genomic DNA, insertion of amplified sequences into pcDNA3.1, and sequencing of randomly picked colonies from transformed *E. Coli*.

### Fc-fusion proteins

cDNA encoding Tie2 ectodomain fusion proteins were constructed by ligating cDNA encoding wild-type or mutant Tie2 ectodomain (residues 1–442) with a GS4 linker, fragment of human Fc immunoglobulin domain, and C-terminal His6 tag. Tie2 ectodomain mutants were created from wild-type human Tie2 ectodomain by site-directed mutagenesis using the QuikChange protocol (Agilent Technologies). All constructs were sequenced to confirm desired mutations.

For protein expression, cDNA encoding fusion proteins in mammalian expression plasmids were transfected into Hek293 cells using polyethylenimine (25). Expressed protein was allowed to accumulate in culture medium for approximately 3 days and media was then clarified by centrifugation and filtration. His6-tagged proteins were recovered by nickel chromatography and, after extensive column washing, eluted with imidazole. Purified proteins were transferred to Tris-buffered saline (TBS; 25 mM Tris, 150 mM NaCl) containing 10% glycerol using Zeba columns (Thermo Fisher). Purity of proteins was assessed by SDS–polyacrylamide gel electrophoresis and Coomassie staining, protein concentrations were determined by Bradford assay, and fusion proteins stored at 4 °C.

### Binding assays

Binding assays were performed by ELISA. Assays were performed in Maxisorp plates (Nunc) in which recombinant Ang1 or Ang2 was immobilized and free binding sites blocked by incubation of wells with TBS containing 0.1% (v/v) Triton-X100 and 5% (w/v) milk powder (Tesco). Fusion proteins were allowed to bind for 1 h, following which wells were washed extensively. Bound Tie2 was detected with anti-Tie2 ectodomain antibodies followed by horseradish-peroxidase-conjugated secondary antibodies and colorimetric quantitation.

### Data analysis

ELISA data were fitted to saturation binding curves using Graphpad Prism 8. Concentrations of ectodomain required for 50% maximal binding were derived from binding curves. In each assay binding of wild-type Tie2 ectodomain was measured in parallel in order to allow calculation of maximal binding of mutant Tie2 as a fraction of maximal wild-type binding.

## Data availability

All data are contained in the article.

## Conflict of interest

The authors declare that they have no conflicts of interest with the contents of this article.
